# Clinicopathological features of hepatoid adenocarcinoma and non‐hepatoid adenocarcinoma of the stomach: A systematic review and meta‐analysis

**DOI:** 10.1002/cam4.70130

**Published:** 2024-08-26

**Authors:** Qi Ling, Han‐Lin Liu, Shi‐Ting Huang, Li‐Fei Sun, Wei‐Wei Wu, Valentin Kudriashov, Kai Liu, Kun Yang, Jian‐Kun Hu, Wei‐Han Zhang

**Affiliations:** ^1^ Department of General Surgery, Gastric Cancer Center West China Hospital, Sichuan University Chengdu Sichuan China; ^2^ West China School of Medicine, Sichuan University Chengdu Sichuan China; ^3^ Department of Obstetrics and Gynecology West China Second University Hospital of Sichuan University Chengdu Sichuan China; ^4^ Key Laboratory of Birth Defects and Related Diseases of Women and Children (Sichuan University), Ministry of Education Chengdu Sichuan China; ^5^ Department of Anesthesiology West China Hospital, Sichuan University Chengdu Sichuan China

**Keywords:** clinicopathological features, Hepatoid adenocarcinoma, non‐hepatoid adenocarcinoma, prognosis

## Abstract

**Background:**

Hepatoid adenocarcinoma of the stomach (HAS) is an extremely rare and unique malignant gastric tumor with a significantly worse prognosis than non‐hepatoid adenocarcinoma of the stomach (non‐HAS). The present study explored the clinicopathological features of HAS and non‐HAS patients to provide insights into HAS treatment strategies.

**Methods:**

From December 26, 2023, we performed a comprehensive search of the PubMed, Web of Science, Cochrane Library, and Embase.com databases for relevant studies. Two authors independently screened the studies, evaluated their quality, extracted data, and performed the analyses. This study was registered with PROSPERO on January 2, 2024.

**Results:**

Nine retrospective studies were included for analysis after screening 833 articles. A total of 350 and 924 patients were enrolled in the HAS and non‐HAS groups, respectively. While no significant differences were observed in age, sex, tumor size, T3 or T4 stage, and N2 or N3 stage between the two groups, the HAS group exhibited higher rates of lymph node metastasis (OR = 1.93, 95% CI: 1.19–3.13, *p* = 0.007), liver metastasis (OR = 3.45, 95% CI: 2.26–5.28, *p* < 0.001), and vascular invasion (OR = 2.76, 95% CI: 2.05–3.71, *p* < 0.001). Additionally, the HAS group had lower 3‐year survival rates (HR = 2.35, 95% CI: 1.70–3.25, *p* < 0.001) and 5‐year survival rates (HR = 3.63, 95% CI: 1.49–8.88, *p* = 0.005), but lower rates of lymphatic permeation (OR = 0.68, 95% CI: 0.47–0.99, *p* = 0.040).

**Conclusion:**

Based on the current clinical evidence, patients with HAS present distinct clinicopathological features, greater invasiveness, and poorer prognosis than non‐HAS patients. Further research is warranted to develop optimal treatment strategies for HAS.

## 1 INTRODUCTION

Hepatoid adenocarcinoma is an extrahepatic tumor that is similar to hepatocellular carcinoma, and it is characterized by its rarity and unique features, including the production of alpha‐fetoprotein (AFP).^1^, ^2^ Hepatoid adenocarcinoma may occur in various organs, including the stomach,^3^ duodenum,^4^ esophagus,^5^, ^6^ jejunum,^7^ colon,^8^ pancreas,^9^, ^10^, ^11^ peritoneum,^12^ gallbladder,^13^ ovary,^14^ lung,^15^ and uterus.^16^ Since its first description by Ishikura et al.^1^ in 1985 as gastric cancer with hepatic differentiation, the stomach has been the most common site for hepatoid adenocarcinoma detection. The annual incidence rate of hepatoid adenocarcinoma of the stomach (HAS) ranges between 0.58 and 0.83 per million residents.^17^, ^18^ Inoue et al.^19^ suggested that HAS accounted for 0.38%–1.6% of all gastric cancer patients, but Chang et al.^20^ reported its incidence as 1.3%–15% of all gastric cancer cases.

The clinical symptoms of HAS resemble common gastric cancer (CGC), which typically presents with upper gastrointestinal discomfort and a lack of specific clinical symptoms. An increase in serum AFP may be a strong indicator of HAS, but it is not necessarily a definitive marker.^21^, ^22^ The current diagnostic criteria for HAS generally follow the criteria outlined in the 2019 WHO classification of digestive system tumors. These criteria focus on the presence of hepatoid components within tumor tissues irrespective of the proportion of hepatoid components and serum AFP levels.^23^ However, HAS exhibits a higher degree of malignancy, which is characterized by rapid progression, a high propensity for liver metastasis, and a poor prognosis.^24^, ^25^ Most clinical studies on HAS are limited to case reports, case series, or small‐sample studies, which results in a lack of consensus on a standardized diagnosis and treatment strategies. In contrast, most case reports suggest that HAS patients have a poorer prognosis than non‐HAS patients.^7^, ^17^, ^26^ However, a minority of studies report no significant difference in prognosis between HAS patients and non‐HAS patients.^27^, ^28^

Therefore, we performed the present study to systematically analyze the clinicopathological features and prognosis of HAS patients compared to non‐HAS patients to provide insights into the treatment of HAS and contribute to the development of more effective therapeutic strategies for this rare and aggressive malignancy.

## 2 MATERIALS AND METHODS

Adhering to the Preferred Reporting Items for Systematic Reviews and Meta‐Analyses (PRISMA) statement 2020^29^, ^30^ criteria was imperative in this systematic review and meta‐analysis and AMSTAR (Assessing the methodological quality of systematic reviews)^31^ Guidelines was used for quality evaluation. As part of our project, we preregistered the study in PROSPERO on January 2, 2024.

### 2.1 Literature search

Relevant research was identified via systematic searches of the PubMed, Cochrane Library, Web of Science, and Embase.com databases. Modifications were made to the subject terms and free words to optimize the terms for the individual databases. The search strategy for PubMed was (((“Stomach Neoplasms”[Mesh]) OR (((((Gastric Neoplasms[Title/Abstract]) OR (Stomach Cancers[Title/Abstract])) OR (Gastric Cancers[Title/Abstract])) OR (gastric carcinomas[Title/Abstract])) OR (gastric tumors[Title/Abstract])))) AND (hepatoid[Title/Abstract]). Manual retrieval of references included in the literature was performed to identify any missing literature from the initially retrieved. The final search was conducted on December 26, 2023.

### 2.2 Inclusion and exclusion

In this systematic review, the following inclusion criteria were used: (1) pathologically confirmed cases of HAS or non‐hepatoid adenocarcinoma of the stomach (non‐HAS); (2) patients who underwent surgery; and (3) studies that compared the differences between HAS patients and non‐HAS patients. The following exclusion criteria were used: (1) studies that lacked non‐HAS or CGC patients as controls; (2) studies with insufficient patient‐related information recorded; and (3) non‐English articles published in full.

### 2.3 Literature screening

After the initial search was completed, duplicate articles were automatically removed, and both authors independently reviewed all of the articles. Initially, articles that potentially met the inclusion criteria were identified based on their titles and abstracts. The full texts of the relevant studies were subsequently obtained, and a manual screening process was performed by the two authors to determine inclusion or exclusion based on the predefined criteria. Any disagreements were resolved via team discussion and verified by a third reviewer to ensure consensus. Each author contributed to the project supervision.

### 2.4 Data extraction

The data were initially extracted independently by two authors using the same table to compile fundamental information from the articles. The extracted information included title, author, type of study, year of publication, patient age, sex composition, tumor size, lymphatic permeation, venous invasion, lymph node metastasis, liver metastasis, survival outcomes, and other relevant data. Survival curves were analyzed to extract information on overall survival (OS) and time‐outcome events. In cases where the necessary information was lacking in the article, we contacted the corresponding author to collect the data. Any discrepancies were resolved by a third reviewer.

### 2.5 Quality assessment

Two authors independently assessed the methodological quality of the nine retrospective studies and resolved any discrepancies via consultation. The Newcastle‐Ottawa Quality Assessment Scale (NOS)^32^ was used to evaluate the quality of the studies, which included criteria such as selection of the study groups (0–4 stars), comparability between groups (0–2 stars), and ascertainment of the exposure or outcome of interest (0–3 stars), for a total of eight categories. Additional stars were awarded for higher‐quality selection and exposure or outcome measurement, with a maximum of one star for each criterion and up to two stars for intergroup comparability. Finally, based on the total number of stars awarded, the studies were categorized as high‐quality (6–9 stars) or low‐quality (0–5 stars).

### 2.6 Statistical analysis

Heterogeneity among the included studies was assessed using *I* ^2^ and Q statistics. Significant heterogeneity was defined as *I* ^2^ > 50% or *p* < 0.1, considering variations in research characteristics, such as patient inclusion and exclusion criteria, treatment strategies, and medical conditions. In cases of significant heterogeneity, the random‐effects model was used; otherwise, the fixed‐effects model was used. All effect sizes are reported with 95% confidence intervals (CIs). Sensitivity analyses were performed using fixed‐effects and random‐effects models to assess the potential impact of model selection on the meta‐analysis outcomes. Meta‐analyses were performed using RevMan 5.4.1, Engauge Digitizer, and R statistical software (Version 4.2.2). Statistical significance was defined as *p* < 0.05.

## 3 RESULTS

### 3.1 Study selection

**FIGURE 1:**
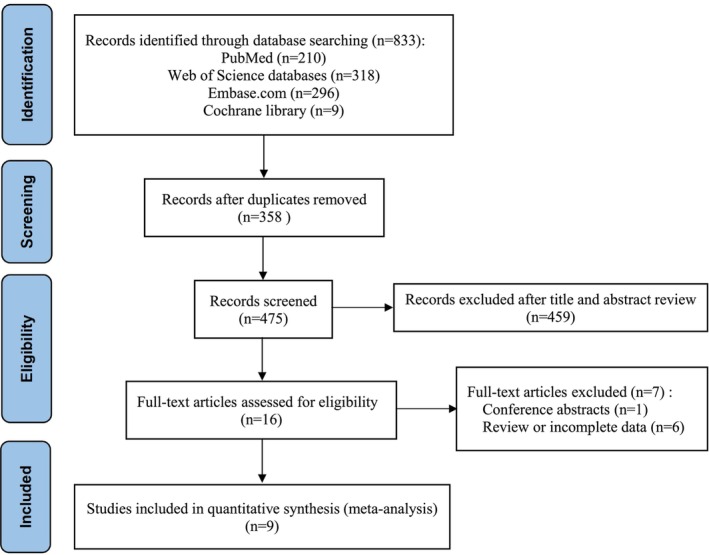
PRISMA selection flow diagram.

A total of 833 potentially relevant studies were identified via the searches of four databases. After deduplication, 475 unique articles remained. The screening of the titles and abstracts of these articles led to the exclusion of 459 studies that did not meet the inclusion criteria. The full texts of the remaining 16 articles were reviewed, which resulted in the exclusion of six studies due to insufficient data or inadequate literature review. Additionally, one conference abstract was excluded. The manual search of the reference lists did not yield any additional studies. Ultimately, nine studies^21^, ^22^, ^24^, ^27^, ^28^, ^33^, ^34^, ^35^, ^36^ that met all of the inclusion criteria were included in the meta‐analysis. Figure 1 shows the flowchart of the literature review process.

### 3.2 Study characteristics

Table 1 provides the comprehensive details of the nine included studies, all of which were retrospective studies performed in China and Japan. These studies collectively involved 350 patients diagnosed with HAS and 924 patients diagnosed with non‐HAS. The methodological quality of the studies was assessed using the NOS, and three studies were allocated six stars, four studies received seven stars, and two studies received eight stars, which indicated that all of the studies were high quality.

**TABLE 1:** Basic characteristics of included studies.

Author	Year	Country	Study design	Time period of patients	Experimental group (*N*)	Control group (*N*)	NOS
Nagai et al.^24^	1993	Japan	R	1962–1991	28	22	6
Kumashiro et al.^33^	2007	Japan	R	1962–2005	23	41	7
Gao et al.^21^	2007	China	R	2001–2003	6	30	6
Liu et al.^34^	2010	China	R	1996–2007	45	225	6
Osada et al.^27^	2014	Japan	R	1979–2013	45	47	7
Fu et al.^35^	2019	China	R	2010–2017	11	38	8
Zhou et al.^28^	2020	China	R	2009.11–2018.12	55	110	7
Huang et al.^22^	2021	China	R	2013.2–2021.2	47	141	8
Jiang et al.^36^	2022	China	R	2009.1–2020.6	90	270	7

Abbreviations: NOS, The Newcastle Ottawa Quality Assessment Scale; R, Retrospective study.

### 3.3 General clinicopathological features

**FIGURE 2:**
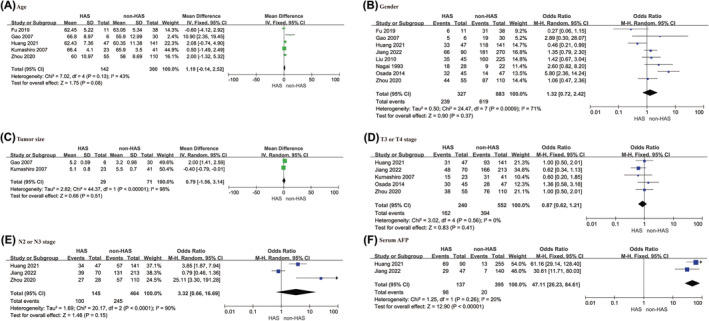
General clinicopathological features: (A) Age; (B) Gender; (C) Tumor Size; (D) T3 or T4 stage; (E) N3 or N4 stage; (F) Serum AFP.

Five articles^21^, ^22^, ^28^, ^33^, ^35^ reported the age of the patients in groups, with an aggregated mean difference (MD) value of 1.19 (95% CI: −0.14–2.52, *p* = 0.080), which indicated no statistically significant difference. No significant heterogeneity was observed (*I* ^2^ = 43%, *p* = 0.130), and the sensitivity analysis confirmed the stability of the findings. The sex composition of the patients was reported in eight studies,^21^, ^22^, ^24^, ^27^, ^28^, ^34^, ^35^, ^36^ with an odds ratio (OR) value of 1.32 (95% CI: 0.72–2.42, *p* = 0.370), which suggests no substantial difference. Tumor size data were available in two articles,^21^, ^33^ which yielded an OR value of 0.79 (95% CI: −1.56–3.14, *p* = 0.510). Five articles^22^, ^27^, ^28^, ^33^, ^36^ reported the T stage, which indicated no significant difference in the T3 or T4 stage (OR = 0.87, 95% CI: 0.62–1.21, *p* = 0.410). N stage was reported in three articles,^22^, ^28^, ^36^ and there was no significant difference in N2 or N3 stage (OR = 3.32, 95% CI: 0.66–16.69, *p* = 0.150). Two studies^22^, ^36^ reported increased serum AFP levels, with a significant increase observed in the HAS group (OR = 47.11, 95% CI: 26.23–84.61, *p* < 0.001). Additionally, Fu et al.^35^ reported a significant difference in the serum AFP levels in patients with HAS (*p* < 0.01). These results are shown in Figure 2, and the sensitivity analysis results are provided in Figure S1.

**FIGURE 3:**
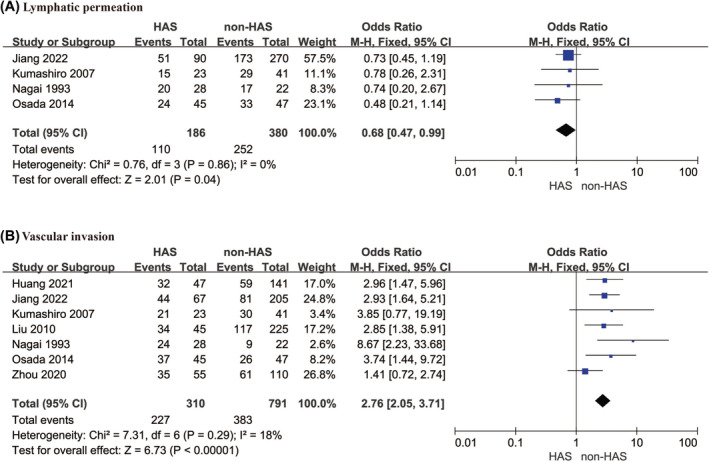
Lymphatic permeation and vascular invasion: (A) Lymphatic permeation; (B) Vascular invasion.

Four articles^24^, ^27^, ^33^, ^36^ reported lymphatic permeation, with 186 and 380 patients in the HAS and non‐HAS groups, respectively. The final analysis revealed an OR of 0.68 (95% CI: 0.47–0.99, *p* = 0.040), which indicated that the degree of lymphatic permeation in the HAS group was lower than that in the non‐HAS group. Sensitivity analysis confirmed the stability of the findings, and no significant heterogeneity was observed (*I* ^2^ = 0%, *p* = 0.860). Vascular invasion was reported in seven articles,^22^, ^24^, ^27^, ^28^, ^33^, ^34^, ^36^ with 310 and 791 patients in the HAS and non‐HAS groups, respectively. The results revealed an OR of 2.76 (95% CI: 2.05–3.71, *p* < 0.001), which indicated that vascular invasion was significantly greater in the HAS group compared to the non‐HAS group. No significant heterogeneity was observed (*I* ^2^ = 18%, *p* = 0.290). Figure 3 shows these results, and the sensitivity analysis results are provided in Figure S2.

### 3.4 Lymph nodes metastasis and distant metastasis

Six articles^21^, ^24^, ^27^, ^33^, ^34^, ^36^ reported the lymph node metastasis ratio, which yielded an OR value of 1.40 (95% CI: 0.76–2.57, *p* = 0.280) and suggested no significant difference between the two groups. However, the sensitivity analysis revealed significant heterogeneity in the study by Jiang et al.^36^ (*I* ^2^ = 53%, *p* = 0.060). Therefore, this study was excluded from the analysis. Subsequent analysis of the remaining five articles^21^, ^24^, ^27^, ^33^, ^34^ included 147 patients in the HAS group and 365 patients in the non‐HAS group. The final results indicated an OR value of 1.93 (95% CI: 1.19–3.13, *p* = 0.007), which suggested that lymph node metastasis was significantly higher in the HAS group than in the non‐HAS group.

Simultaneous or metachronous liver metastases were reported in six studies.^21^, ^24^, ^27^, ^33^, ^34^, ^36^ The results revealed an OR value of 6.06 (95% CI: 2.09–17.60, *p* < 0.001), which suggested a higher rate of liver metastasis in patients with HAS. However, the sensitivity analysis revealed significant heterogeneity in the study by Liu et al.^34^ (*I* ^2^ = 80%, *p* < 0.001), and this study was excluded from the analysis. After this study was excluded, the final analysis included 185 patients in the HAS group and 401 patients in the non‐HAS group. The results revealed an OR of 3.45 (95% CI: 2.26–5.28, *p* < 0.001), which indicated a higher rate of liver metastasis in the HAS group than in the non‐HAS group. In addition, Liu et al.^34^ reported simultaneous and heterotemporal liver metastasis rates of 8.9% and 73.2%, respectively, in the HAS group and 1.8% and 9.9%, respectively, in the non‐HAS group. The results indicated that the rate of liver metastasis was significantly higher in the HAS group than in the non‐HAS group. Moreover, the time from surgery to heterochronic liver metastasis in the HAS group was significantly shorter than in the non‐HAS group (6.0 ± 2.7 vs. 20.2 ± 10.8 months, *p* < 0.01).

**FIGURE 4:**
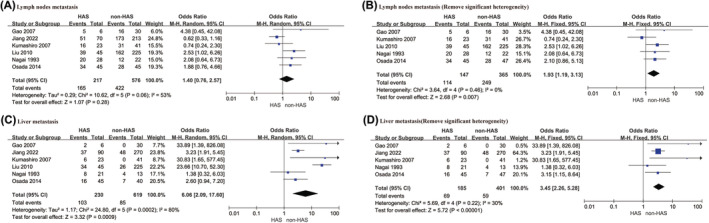
Lymph nodes metastasis and liver metastasis: (A) Lymph nodes metastasis; (B) Lymph nodes metastasis (Remove significant heterogeneity): (C) Liver metastasis; (D) Liver metastasis (Remove significant heterogeneity).

Two articles^33^, ^36^ reported metastases to other organs. Kumashiro et al.^33^ reported one case of splenic metastasis and one case of peritoneal metastasis in patients with HAS and two cases of peritoneal metastasis and one case of lung metastasis in patients with non‐HAS. Jiang et al.^36^ reported that most of the metastatic sites (97.3%) were liver metastases in the HAS group, whereas in the non‐HAS group, peritoneal metastases accounted for 35.3%, liver metastases only accounted for 34.3%, and multiple systemic metastases occurred at other sites. Because of insufficient data, a meta‐analysis of metastases to other organs was not possible. These findings are summarized in Figure 4, and the sensitivity analysis results are shown in Figure S3.

### 3.5 Overall survival

**FIGURE 5:**
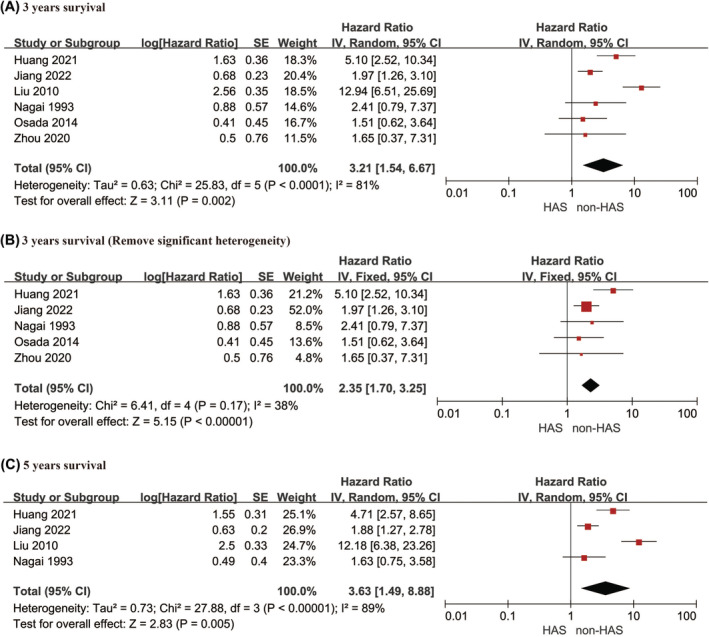
Overall survival: (A) 3‐year survival; (B) 3‐year (Remove significant heterogeneity); (C) 5‐year survival.

Three years survival outcomes were reported in six studies,^22^, ^24^, ^27^, ^28^, ^34^, ^36^ and the results revealed an HR value of 3.21 (95% CI: 1.54–6.67, *p* = 0.002), which indicated a lower 3‐year survival rate for patients in the HAS group. Sensitivity analysis revealed significant differences in the study by Liu et al.^34^ (*I* ^2^ = 81%, *p* < 0.001), which led to its exclusion from further analyses. After exclusion, the final HR value was 2.35 (95% CI: 1.70–3.25, *p* < 0.001), which confirmed a significantly lower OS for HAS patients than for non‐HAS patients. Additionally, four articles^22^, ^24^, ^34^, ^36^ reported a 5‐year survival rate with an HR value of 3.63 (95% CI: 1.49–8.88, *p* = 0.005), which indicated a lower 5‐year survival rate for patients in the HAS group. However, the sensitivity analysis revealed strong heterogeneity (*I* ^2^ = 89%, *p* < 0.001). These results are shown in Figure 5, and the sensitivity analysis results are presented in Figure S4.

## 4 DISCUSSION

Given the exceedingly low incidence of HAS and the limited sample size available for analysis, there is little evidence to systematically summarize the clinical characteristics of HAS. The present study compared the clinicopathological features of HAS and non‐HAS cases. The results revealed no substantial differences between the two groups in demographic variables such as age or sex. However, there was a significant increase in lymph node metastasis, liver metastasis, and vascular invasion in the HAS group. Additionally, the OS rate was notably decreased in patients with HAS. Interestingly, lymphatic permeation was higher in the non‐HAS group than in the HAS group. We hypothesized that the reduced lymphatic permeation of HAS compared to non‐HAS in our study may be attributed to metastasis via direct invasion or hematogenous spread in gastric hepatoid adenocarcinoma. Additionally, the expression profiles of microsatellite instability (MSI) and immunogenic tumor cell features may differ in HAS. However, further investigation is needed to confirm the validity of these findings.

The clinical manifestations of HAS patients are generally atypical and similar to the presentations of CGC patients, such as abdominal pain, epigastric discomfort, hematemesis, or black stool.^7^ However, serum AFP is generally high in patients with HAS, but other tumor markers remain normal.^7^ Several of the included studies reported serum AFP levels. Specifically, two studies^22^, ^36^ investigated whether the AFP level increased. The results revealed that the increase in the serum AFP level in the HAS group was statistically significant, which indicated that the serum AFP levels is increased in most patients with HAS, but not in every patient. Previous studies have shown that patients with elevated serum AFP level had a poorer prognosis than patients without elevated serum AFP levels, with AFP levels >300 ng/mL being such a factor.^37^, ^38^ Nevertheless, another report indicated that the prognosis did not significantly differ between individuals with AFP levels >300 ng/mL and patients with AFP levels <300 ng/mL^7^. HAS also has a greater possibility of distant metastasis, with the most common site being the liver, followed by the lungs, peritoneum, spleen, and brain. This observation may also explain the poor prognosis of HAS.

Pathological diagnosis is the gold standard for the diagnosis of HAS. Macroscopically, most patients present with Borrmann type III, with poor differentiation. Microscopically, HAS is characterized by tumor features resembling hepatoid adenocarcinoma, as observed via hematoxylin and eosin (HE) staining. It consists of large eosinophilic cells that resemble hepatocellular carcinoma in shape, are arranged in trabecular or solid nest‐like patterns and are separated by sinusoidal vascular channels.^28^, ^37^, ^39^ Recent studies demonstrated that HAS exhibits specific immunohistochemical characteristics, including high levels of mismatch repair (MMR) protein proficiency, positive AFP or spalt‐like transcription factor 4 (SALL4), overexpression of HER2, and negative Epstein–Barr virus‐encoded small RNA (EBER) expression. Negative EBER and MMR profiles indicate the molecular features of HAS, whereas positive AFP or SALL4 expression aids in its diagnosis.^40^ For the molecular characteristics, TP53 is the most common gene mutation observed in HAS tumor samples, but the CD3EAP, RPTOR, and CEBPA are frequently mutated.^2^, ^40^, ^41^

HAS exhibits a higher incidence of metastasis and is often diagnosed at later stages with increased lymph node involvement and a higher frequency of liver metastasis. Conversely, non‐HAS patients commonly presents with liver metastases. Although peritoneal metastases are also prevalent, they are less dominant than liver metastases in patients with HAS. However, liver metastases in patients with HAS may be classified as simultaneous or post‐operative. Liu et al.^34^ suggested that patients in the HAS group had higher rates of simultaneous and heterochronous liver metastases, and the time from surgery to liver metastases in the HAS group was shorter. Liu et al. reported that among 58 patients with HAS whose initial site of recurrence was identified, the highest proportion of recurrences occurred in the liver (36.2%), followed by multiple systemic metastases (29.3%).^42^ A recent study revealed that frequent alterations in the signaling pathways regulating stem cell pluripotency ware significantly associated with liver metastasis in HAS. These alterations predominantly involve CDK12, MYC, OCT4‐pg1, HUWE1, and MYCBP2.^36^ This study suggested that overactivation of the MYC functional axis induce hepatoid differentiation by influencing stem cell pluripotency, which potentially contributing to the poor prognosis of HAS patients. Another study identified isolated portal vein tumor thrombosis as a risk factor for liver metastasis in patients with HAS.^43^ Therefore, further research is warranted to elucidate the specific mechanisms underlying liver metastases.

In terms of treatment, there remains a dearth of specialized therapeutic approaches for HAS, with current protocols largely mirroring the treatment protocols used for non‐HAS patients, which predominantly rely on surgery‐based perioperative comprehensive treatment. However, despite adhering to existing treatment modalities, therapeutic outcomes are suboptimal, with findings consistently indicating a poorer prognosis for HAS patients than for non‐HAS patients.^22^, ^24^, ^34^, ^36^ While a minority of studies failed to identify a significant difference in prognosis between HAS patients and non‐HAS patients,^28^ this result may be attributed to the propensity score matching of clinicopathological data between the two patient groups in these studies, including liver metastasis rates and pathological stages. Conversely, the potential efficacy of drug treatment strategies for HAS warrants further investigation, with recent reports highlighting promising novel targets such as ERBB2, FGFR2, MET, and HGF gene amplification.^44^

The main limitation of the present study is that it included only nine retrospective clinical studies from China and Japan and lacked prospective research. The primary aim of this study was to compare the clinical features of HAS and non‐HAS. Therefore, single‐arm studies were excluded. However, this selection criterion may have affected the robustness of the study and potentially introducing bias. Additionally, only one article in the included literature provided detailed information on the timing of liver metastasis (pre‐ or post‐operative), and further subgroup analysis could not be performed, which may have caused some bias in survival outcomes. In addition, there was considerable heterogeneity in some results, which was addressed using a random‐effects model to account for significant heterogeneity (*I* ^2^ > 50% or *p* < 0.01). However, this approach may still introduce bias into the analysis.

## 5 CONCLUSIONS

The diagnosis and comprehension of HAS have progressed with advancements in pathological and molecular diagnostic techniques. Existing research indicates that HAS presents with advanced staging, poorer prognosis, and a higher risk of liver metastasis than non‐HAS. To enhance the therapeutic outcomes of HAS, further research and a deeper understanding are warranted to devise specialized treatment strategies tailored to this condition.

## AUTHOR CONTRIBUTIONS

**Wei‐Han Zhang:** Conceptualization (lead); data curation (lead); formal analysis (lead); funding acquisition (lead); investigation (lead); methodology (lead); project administration (lead); resources (lead); supervision (lead); validation (lead); writing – original draft (lead). **Qi Ling:** Data curation (lead); formal analysis (supporting); software (lead); validation (supporting); visualization (lead); writing – original draft (lead). **Han‐Lin Liu:** Data curation (supporting); investigation (supporting). **Shi‐Ting Huang:** Investigation (supporting); writing – review and editing (supporting). **Li‐Fei Sun:** Investigation (supporting); writing – review and editing (supporting). **Wei‐Wei Wu:** Funding acquisition (lead); investigation (supporting). **Valentin Kudriashov:** Investigation (supporting); writing – review and editing (supporting). **Kai Liu:** Investigation (supporting); writing – review and editing (supporting). **Kun Yang:** Formal analysis (lead); software (lead); writing – review and editing (supporting). **Jian‐Kun Hu:** Funding acquisition (lead); investigation (equal); methodology (equal); project administration (equal); writing – original draft (lead).

## FUNDING INFORMATION

Medical Science and Technique Project of Health Commission of Sichuan Province (No. 21PJ043). Natural Science Foundation of Sichuan Province (No.23NSFSC1611, No.2023NSFSC1847, No.24ZDYF0399). Sichuan University “From 0 to 1” innovative research project (2023SCUH0057).

## CONFLICT OF INTEREST STATEMENT

The authors declare that they have NO affiliations with or involvement in any organization or entity with any financial interest in the subject matter or materials discussed in this manuscript.

## Supporting information


Data S1:

## Data Availability

On reasonable request, the corresponding author will provide the data that support the conclusions of this study.
