# Supplementary material for: Clinicopathological features of hepatoid adenocarcinoma and non‐hepatoid adenocarcinoma of the stomach: A systematic review and meta‐analysis
Source: Cancer Med. 2024 Aug 26;13(16):e70130. doi: 10.1002/cam4.70130 (PMC11346349; doi:10.1002/cam4.70130)
Supplement: Supplementary file 1 — Data S1: [file CAM4-13-e70130-s001.pdf]

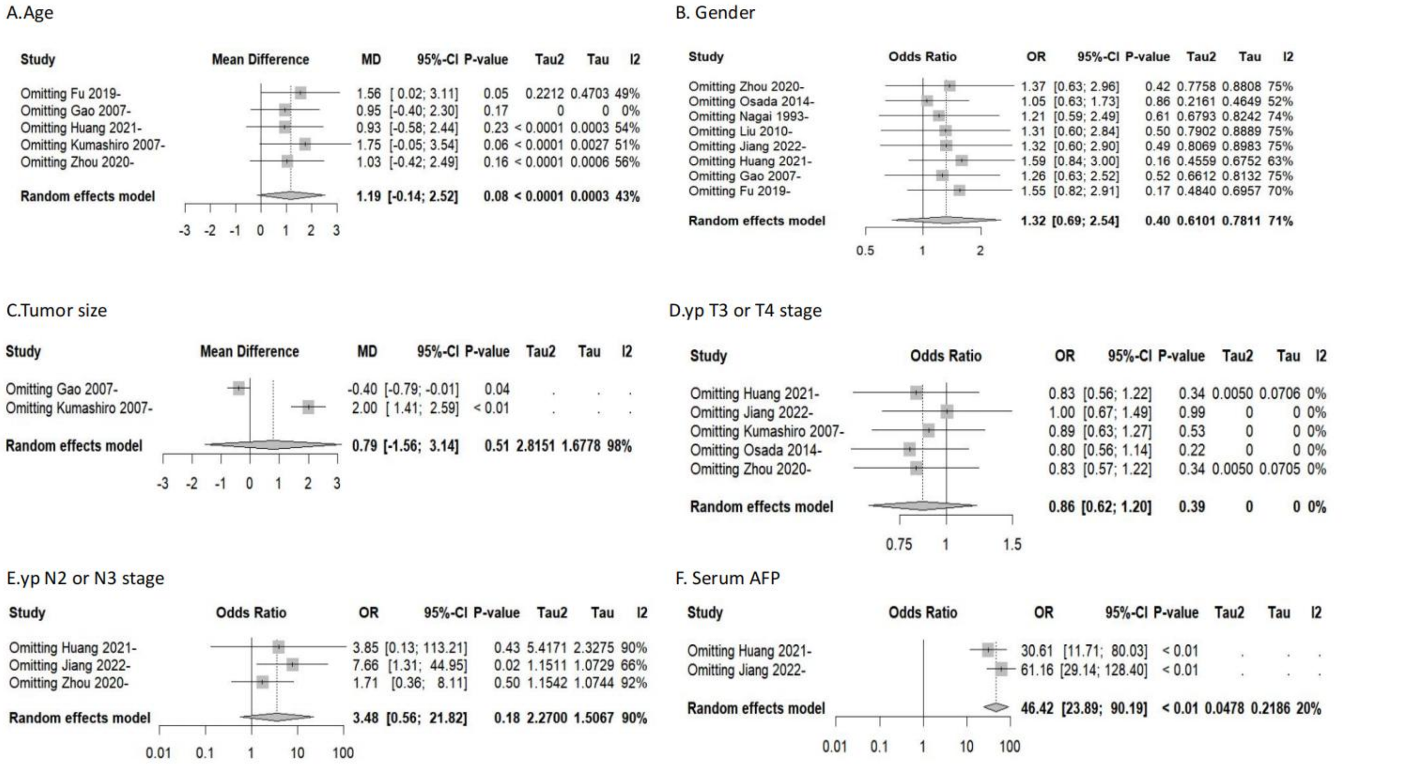

Figure S1. Sensitivity analysis of general clinicopathological features: (A) Age; (B) Gender; (C) Tumor Size; (D) T3 or T4 stage; (E) N3 or N4 stage; (F) Serum AFP.

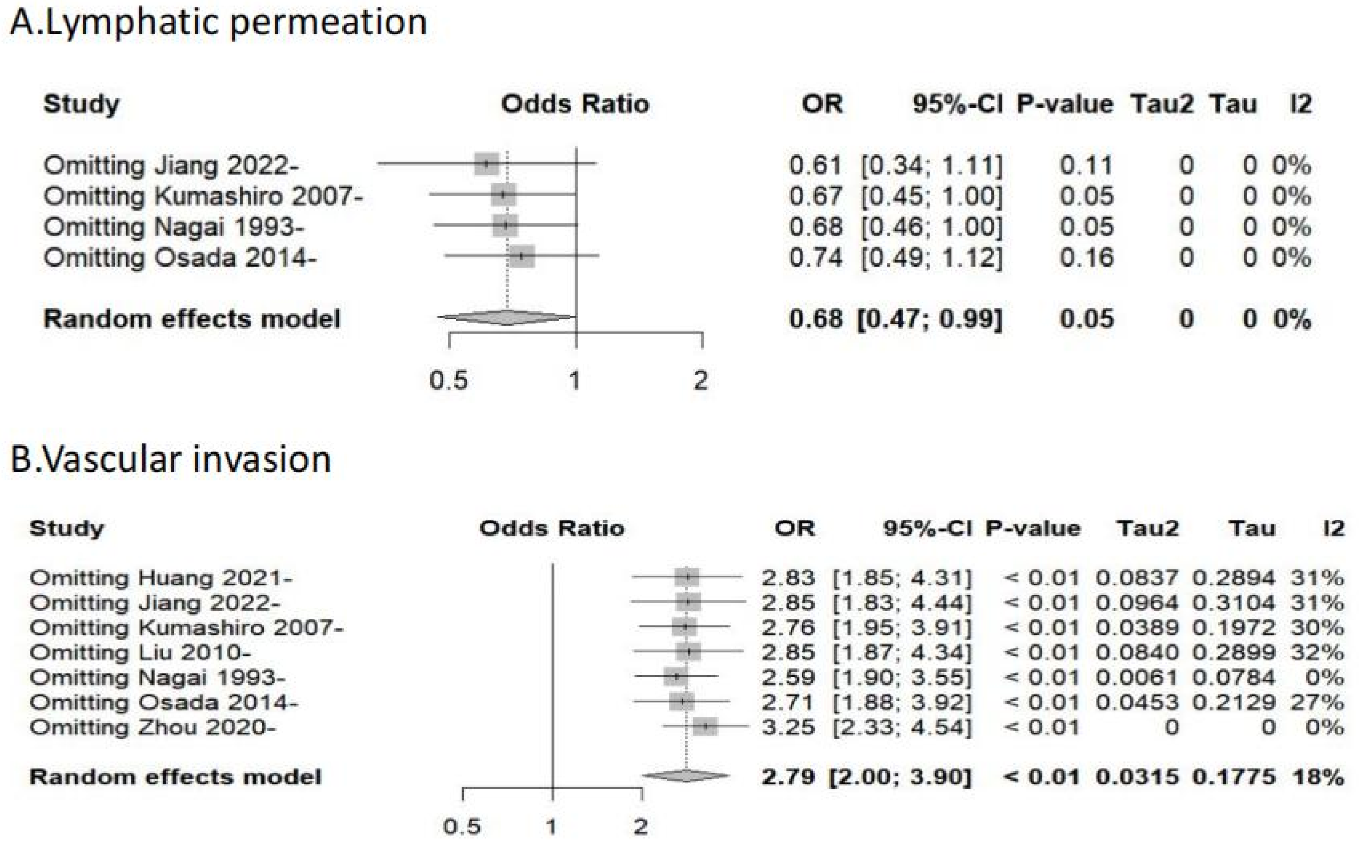

Figure S2. Sensitivity analysis of lymphatic permeation and vascular invasion: (A) Lymphatic permeation; (B) Vascular invasion.

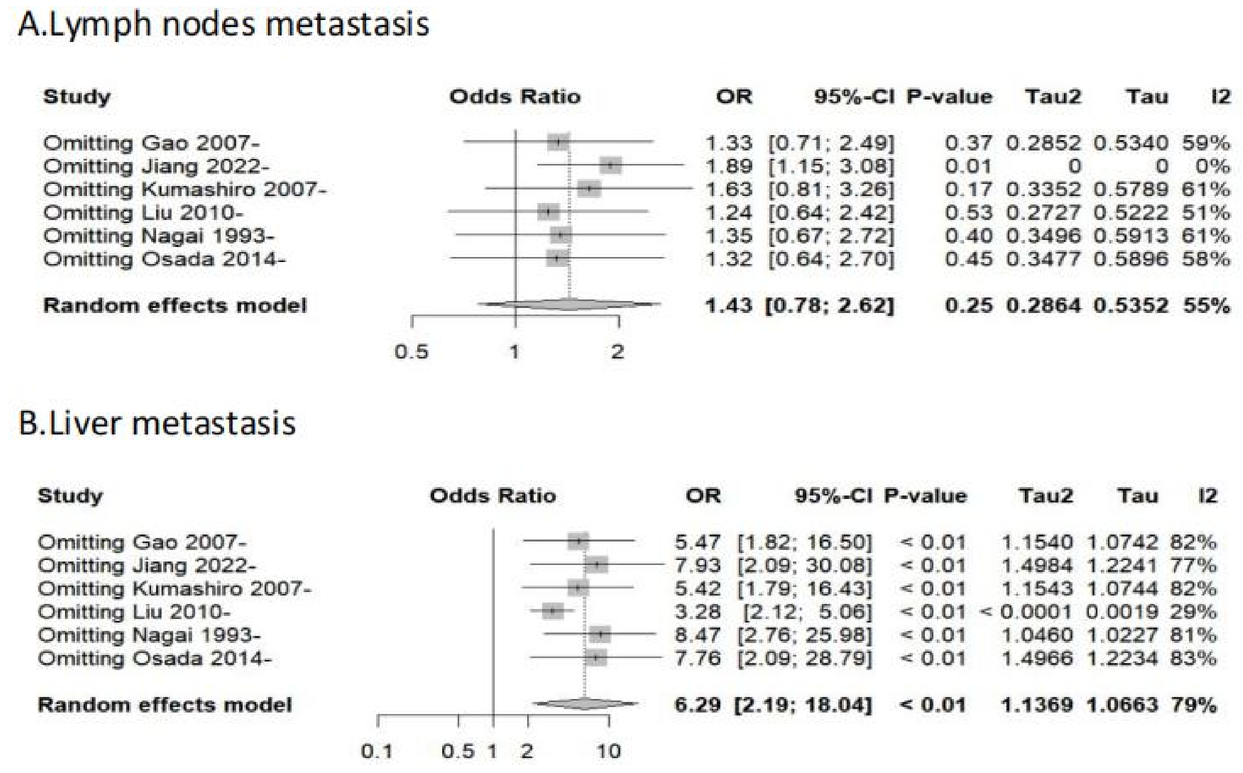

Figure S3. Sensitivity analysis of lymph nodes metastasis and liver metastasis:(A) Lymph nodes metastasis; (B) Liver metastasis.

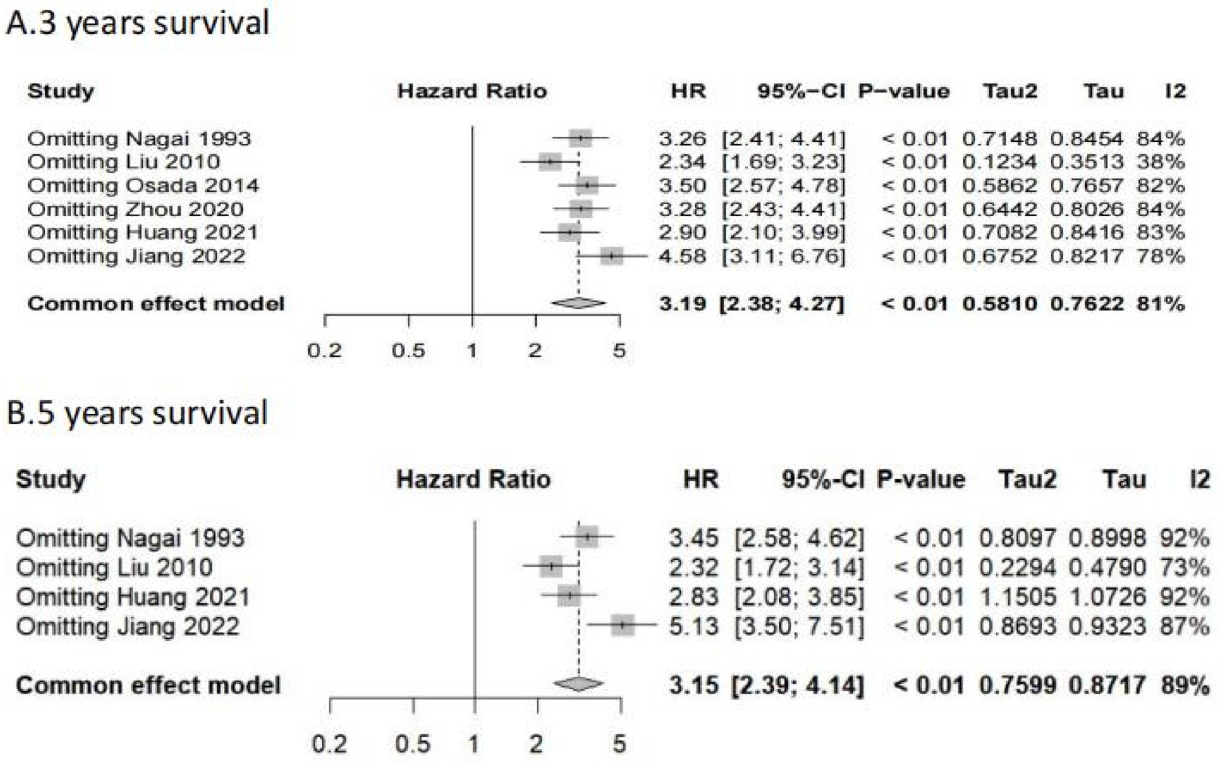

Figure S4. Sensitivity analysis of overall survival: (A) 3 year survival; (B) 5 year survival.
